# Methylation regulates HEY1 expression in glioblastoma

**DOI:** 10.18632/oncotarget.17897

**Published:** 2017-05-16

**Authors:** Andrew J. Tsung, Maheedhara R. Guda, Swapna Asuthkar, Collin M. Labak, Ian J. Purvis, Yining Lu, Neha Jain, Sarah E. Bach, Durbaka V.R. Prasad, Kiran K. Velpula

**Affiliations:** ^1^ Department of Cancer Biology and Pharmacology, University of Illinois College of Medicine at Peoria, Peoria, IL, USA; ^2^ Department of Neurosurgery, University of Illinois College of Medicine at Peoria, Peoria, IL, USA; ^3^ Illinois Neurological Institute, Peoria, IL, USA; ^4^ Department of Pathology, Peoria, IL, USA; ^5^ Department of Microbiology, Yogi Vemana University, Kadapa, India

**Keywords:** glioblastoma, methylation, p53, proliferation, DNMT1

## Abstract

Glioblastoma (GBM) remains one of the most lethal and difficult-to-treat cancers of the central nervous system. The poor prognosis in GBM patients is due in part to its resistance to available treatments, which calls for identifying novel molecular therapeutic targets. In this study, we identified a mediator of Notch signaling, HEY1, whose methylation status contributes to the pathogenesis of GBM. Datamining studies, immunohistochemistry and immunoblot analysis showed that HEY1 is highly expressed in GBM patient specimens. Since methylation status of HEY1 may control its expression, we conducted bisulphite sequencing on patient samples and found that the HEY1 promoter region was hypermethylated in normal brain when compared to GBM specimens. Treatment on 4910 and 5310 xenograft cell lines with sodium butyrate (NaB) significantly decreased HEY1 expression with a concomitant increase in DNMT1 expression, confirming that promoter methylation may regulate HEY1 expression in GBM. NaB treatment also induced apoptosis of GBM cells as measured by flow cytometric analysis. Further, silencing of HEY1 reduced invasion, migration and proliferation in 4910 and 5310 cells. Furthermore, immunoblot and q-PCR analysis showed the existence of a potential positive regulatory loop between HEY1 and p53. Additionally, transcription factor interaction array with HEY1 recombinant protein predicted a correlation with p53 and provided various bonafide targets of HEY1. Collectively, these studies suggest HEY1 may be an important predictive marker for GBM and potential target for future GBM therapy.

## INTRODUCTION

Glioblastoma (GBM) is one of the most difficult cancers to treat, with a very poor prognosis. Even with the advent of temozolomide (TMZ), two-year survival rates have stagnated at 17%, and the incidence of recurrence and chemoresistance to TMZ is high [1]. Current therapeutic strategies attempt to address the uncontrolled proliferation within the tumor's core [2], as well as that occurs in the tumor's periphery [3]. Due to its contribution toward both of these tumor characteristics, the Notch pathway has become an interesting target [4–6]. The Hairy/Enhancer-Of-Split related with YRPW Motif 1 (HEY1) belonging to basic helix-loop-helix transcription factor family that co-activate components of the Notch and its dependent signaling pathways. HEY1 has been found to be expressed within solid CNS tumors [7], and the blockade of HEY1 via antisense oligonucleotide has been shown to inhibit hallmarks of the tumor phenotype *in vitro* [8].

In general, global DNA hypomethylation signals conversion from normal growth patterns to those that are characteristic of tumors: unchecked proliferation, cell migration, and angiogenesis [12]. In such cases, Cytosine-phosphate-Guanine (CpG) islands serve as primary sites of DNA methylation [13]. Sufficient cytosine methylation correlates with the shutdown of a linked promoter and its associated gene [14]. Therapies that exist to target this global hypomethylation observed in cancers include sodium butyrate (NaB), a DNA methylating agent. NaB has the ability to re-methylate previously hypomethylated regions of gene sequence [15]. Furthermore, agents such as NaB have been demonstrated to work in part through the DNA methyltransferase/MAP1 Kinase (DNMT/ERK) axis [16]. DNMTs act to catalyze DNA methylation by transference of a methyl group on cytosine [17]. Additionally, the tumor suppressor gene p53 takes on a mutant form in neural progenitor cells and may give rise to GBM tumors [9]. It has been shown that the efficacy of first-line GBM treatment, temozolomide (TMZ), is largely dependent on p53 status in the tumor [10], where mutant p53 status confers enhanced proliferative abilities in the tumor and resistance to TMZ [11].

The aims of this paper are twofold. First, we demonstrate that hypomethylation of HEY1 contributes to the proliferative properties in GBM. Therapies that re-methylate its promoter region or interfere with the transcription of HEY1 including NaB or HEY1 siRNAs, respectively, can reduce neoplastic phenotypes. Second, we identify targets that interact with and modulate the transcriptional activity of HEY1, including the proposed upstream methyltransferase DNMT1 responsible for epigenetic control, and downstream mutant p53, which may contribute to cancer phenotypes in GBM.

## RESULTS

### HEY1 is expressed in human glioblastoma (hGBM) patient-derived samples

**Figure 1:**
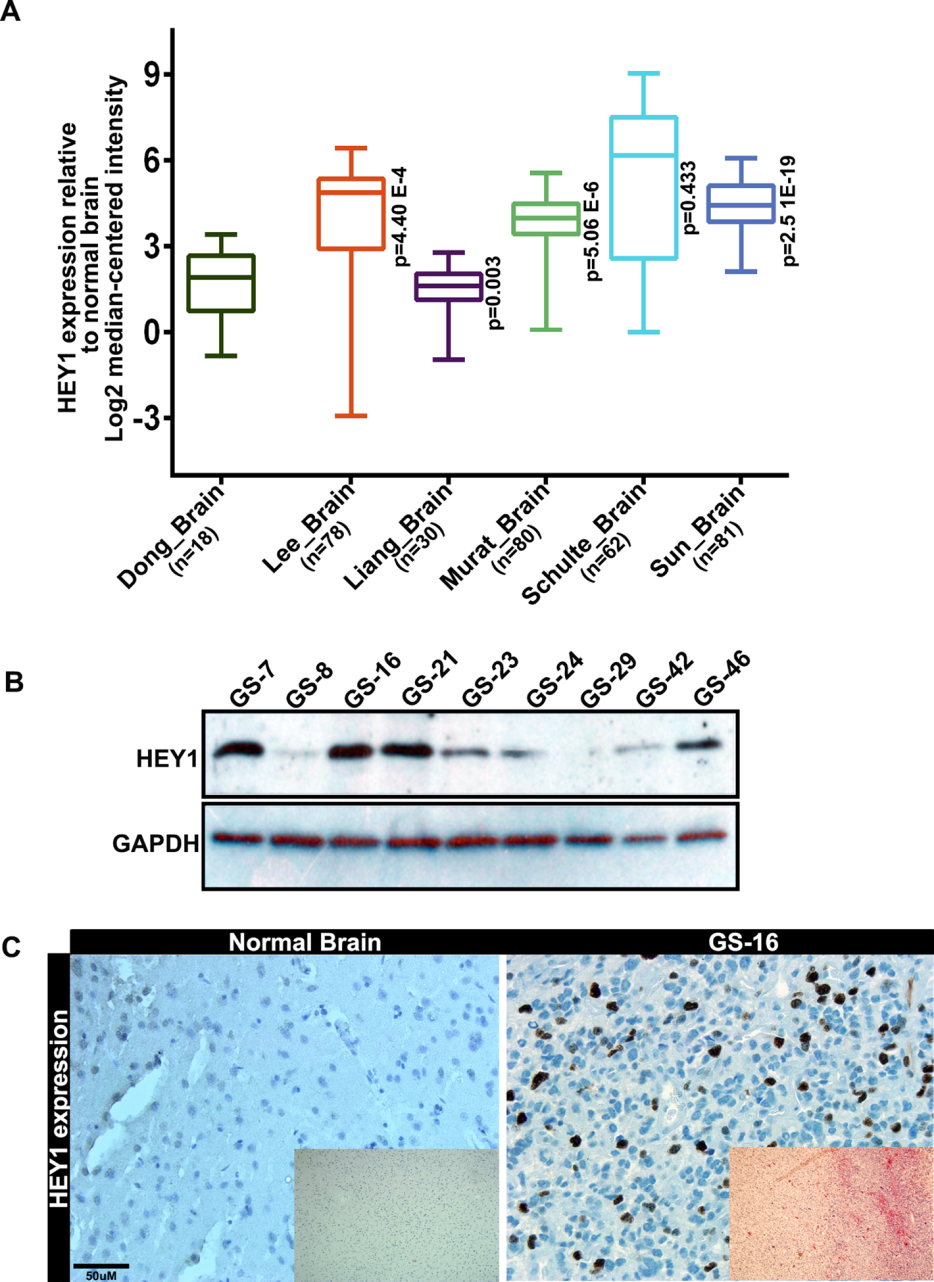
HEY1 is overexpressed in hGBM patient specimens (**A**) Whisker plots of HEY1 gene expression catalogued within the Oncomine^™^ database amongst various large-sample studies (**B**) Immunoblotting analysis GBM samples (GS), (*n* = 9) for detecting HEY1 expression (**C**) Immunohistochemical analysis of normal brain and GS-16 to detect HEY1 using DAB staining (Bar = 50 μM). Inset images represent hematoxylin and eosin staining of the respective specimens.

Increased levels of HEY1 have been previously implicated in GBM and are considered to be an important factor in the formation of GBM [18, 19]. Here, we first assessed if an increase in HEY1 expression was characteristic in GBM patients using data mining approach. Using the Oncomine™ database, we compared the mRNA expression of HEY1 from Dong, Lee, Liang, Murat, Schulte and Sun datasets. Of all the datasets, Schulte dataset showed 9-fold expression increase compared to normal brain, while the other datasets showed a range of 3–6 fold expression increase. Expression levels of HEY1 are compared to that in normal tissues by using its value of log2 median-center intensity (Figure 1A). To further validate the Oncomine findings, we conducted immunoblot analysis on total protein lysates of 9 hGBM surgical biopsy specimens. In the 9 specimen tested, most showed expression of HEY1, indicating its role in hGBM. Interestingly, normal human brain specimen showed no expression of HEY1 (Figure 1B and Supplementary Figure 1). Immunohistochemical analysis on Glioblastoma Specimen 16 (GS-16) showed increased nuclear staining on HEY1 while negligible expression is observed in the normal brain specimen (Figure 1C).

### HEY1 promoter is hypomethylated in GBM

**Figure 2:**
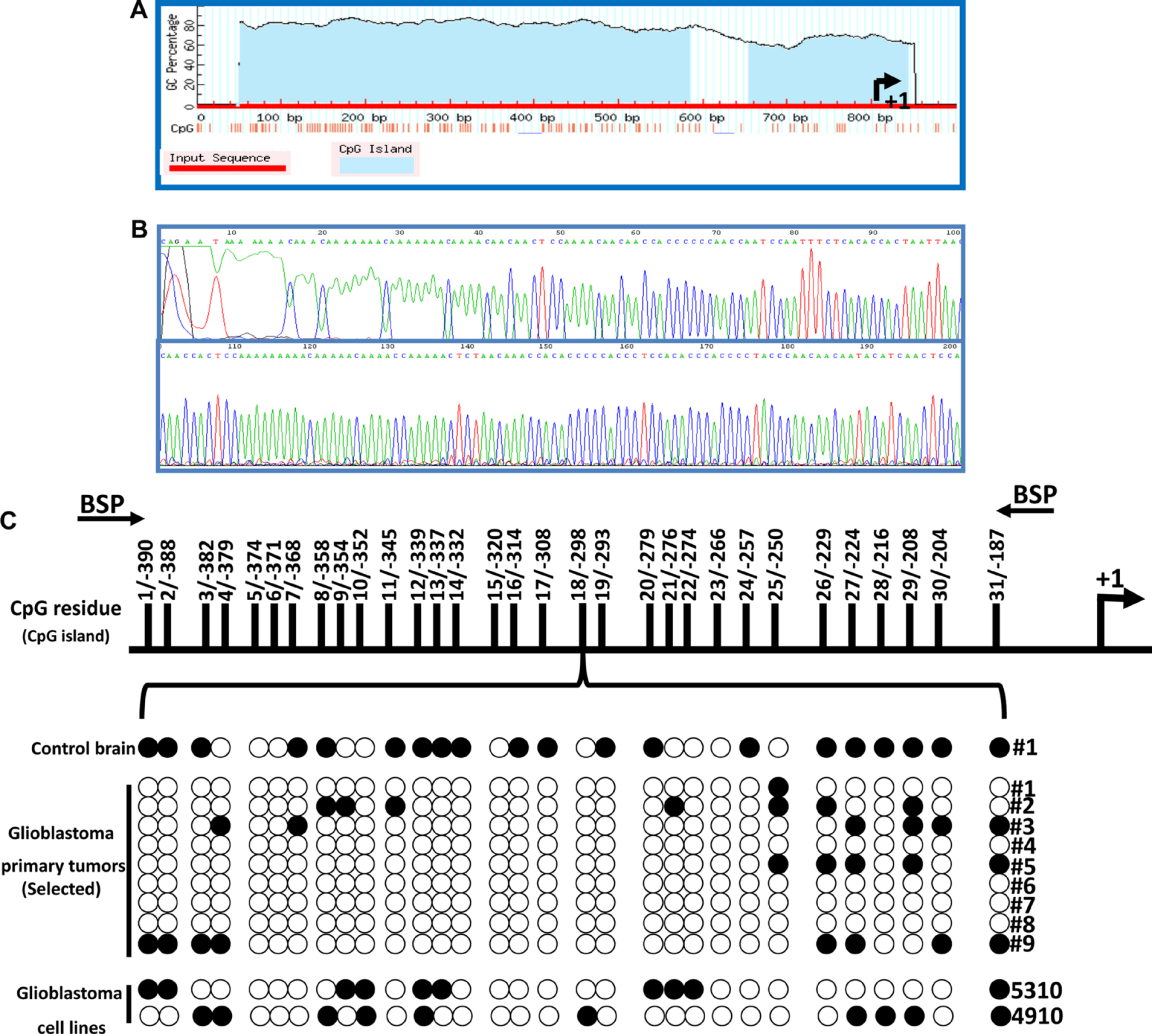
Cytosine-phosphate-Guanine (CpG) islands found on the promoter region of the HEY1 gene take on a demethylated status in GBM (**A**) CpG islands are observed at relatively high percentages in the promoter region (left of arrow) of the *HEY1* gene based upon genomic labeling (**B**) Schematic representation of bisulphite sequencing (**C**) Results of BSP analysis of HEY1 promoter CpG island region. *Top*, schematic representation of the HEY1 promoter highlighting the position of CpG island relative to transcription start site. *Bottom*, representative sequencing results of the BSP products. Areas of methylation are denoted as black circles and areas of demethylation are denoted as white circles in the control and GBM samples’ HEY1 promoter regions.

Adequate methylation of CpG islands within the genome has been implicated in proper neural function and is considered a crucial epigenetic regulatory mechanism [20]. Alterations in methylation status can lead to neoplastic growth and ultimately cancer. To investigate the transcriptional machinery responsible for regulating HEY1 expression, we first identified the HEY1 promoter sequence on chromosome 8 (80677423–80679898), 5000-bp upstream from the translation initiation start site. Figure 2A shows a representation of CpG islands designed using MethPrimer software and Figure 2B shows a schematic representation of bisulphite sequencing. Briefly, bisulphite-modified DNA obtained from nine hGBM specimen, one normal control brain specimen, and cell lines 4910 and 5310 were amplified using BSP primers and sequenced. The methylation status of 31 CpG sites within the CpG island of the HEY1 promoter region (-397 to -187) are shown in Figure 2C. Taken together, we conclude that HEY1 promoter is hypomethylated in hGBM patient specimens and cell lines, while normal brain sample showed dense methylation.

### Influence of sodium butyrate (NaB) on HEY1 expression

**Figure 3:**
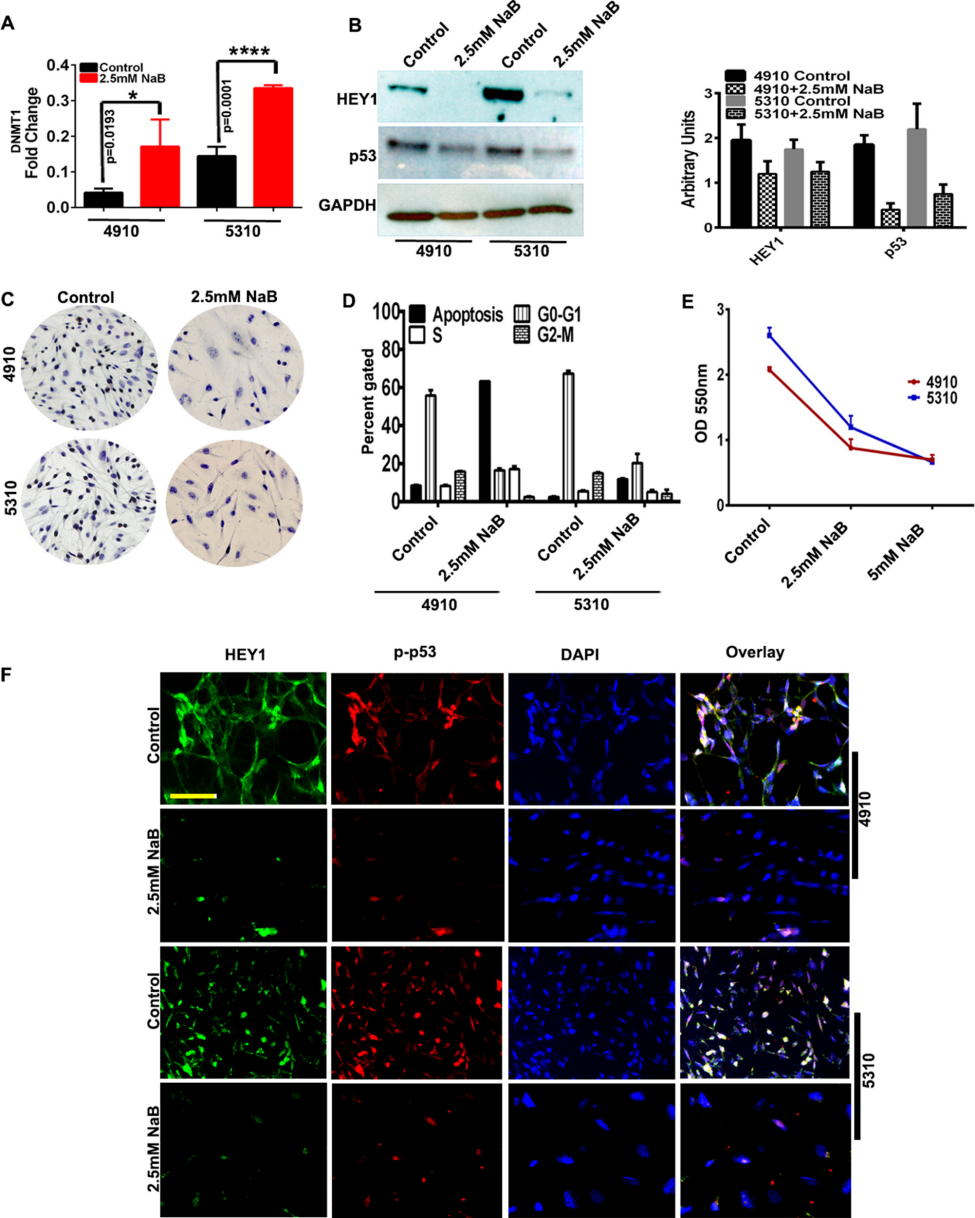
Sodium Butyrate (NaB) inhibits expression of HEY1/p53 *in vitro* (**A**) qRT-PCR demonstrating expression levels of DNA Methyl Transferase 1 (DNMT1) in 4910, 5310 cell lines with control and 2.5mM NaB treated cells (**B**) Immunoblot analysis of HEY1 and p53 in control and 2.5 mM NaB treatment in 4910 and 5310 cells (**C**) DAB staining for control and 2.5 mM NaB treated cells (**D**) Fluorescence-Activated Cell Sorting (FACS) outlines the effect of NaB treatment on cell cycle progression of 4910 and 5310 cells (**E**) MTT assay on 2.5 mM and 5mM NaB treated cells (**F**) Immunocytochemistry demonstrates HEY1 and phospho-p53 co-localization in control cells; NaB treatment reduce this colocalization (Bar = 100 μM).

NaB is a potential therapeutic agent known to methylate DNA [12] and has shown to inhibit growth, proliferation and influence gene expression in glioma cells [21]. As we observed that HEY1 promoter is hypomethylated, we investigated whether NaB could reduce the expression of HEY1 in 4910 and 5310 cells. Both cell lines were treated with 2.5 mM NaB for 24 h. First, we measured the expression levels of DNMT1 using qPCR analysis in 2.5 mM NaB treated cells. In both the treated cell lines, *DNMT1* gene expression is observed to be increased, suggestive of the active methylation induced by 2.5 mM NaB treatment (Figure 3A). Next, using immunoblotting, we observed that the presence of HEY1 and p53 was significantly reduced in cell lines 4910 and 5310, following treatment with 2.5 mM NaB (Figure 3B). DAB immunohistochemistry further confirmed this observation, showing reduced levels of HEY1 with NaB treatment (Figure 3C). Next, FACS analysis showed increased apoptosis with reduced proliferation in the NaB treated 4910 and 5310 cells when compared to their respective controls (Figure 3D). Further, MTT assay conducted using 10,000 cells of 4910 and 5310 with 2.5 mM and 5 mM NaB treatments showed reduced cell proliferation when compared to their respective controls (Figure 3E). Additionally, using immunofluorescence staining, we confirmed that both HEY1 and phospho-p53 interact in glioma cells and that NaB treatment reduced their interaction significantly (Figure 3F). These results suggest that NaB treatment may regulate glioma growth and proliferation by regulating the expression of HEY1 via methylation.

### Silencing HEY1 corroborates NaB effects in glioma cells

**Figure 4:**
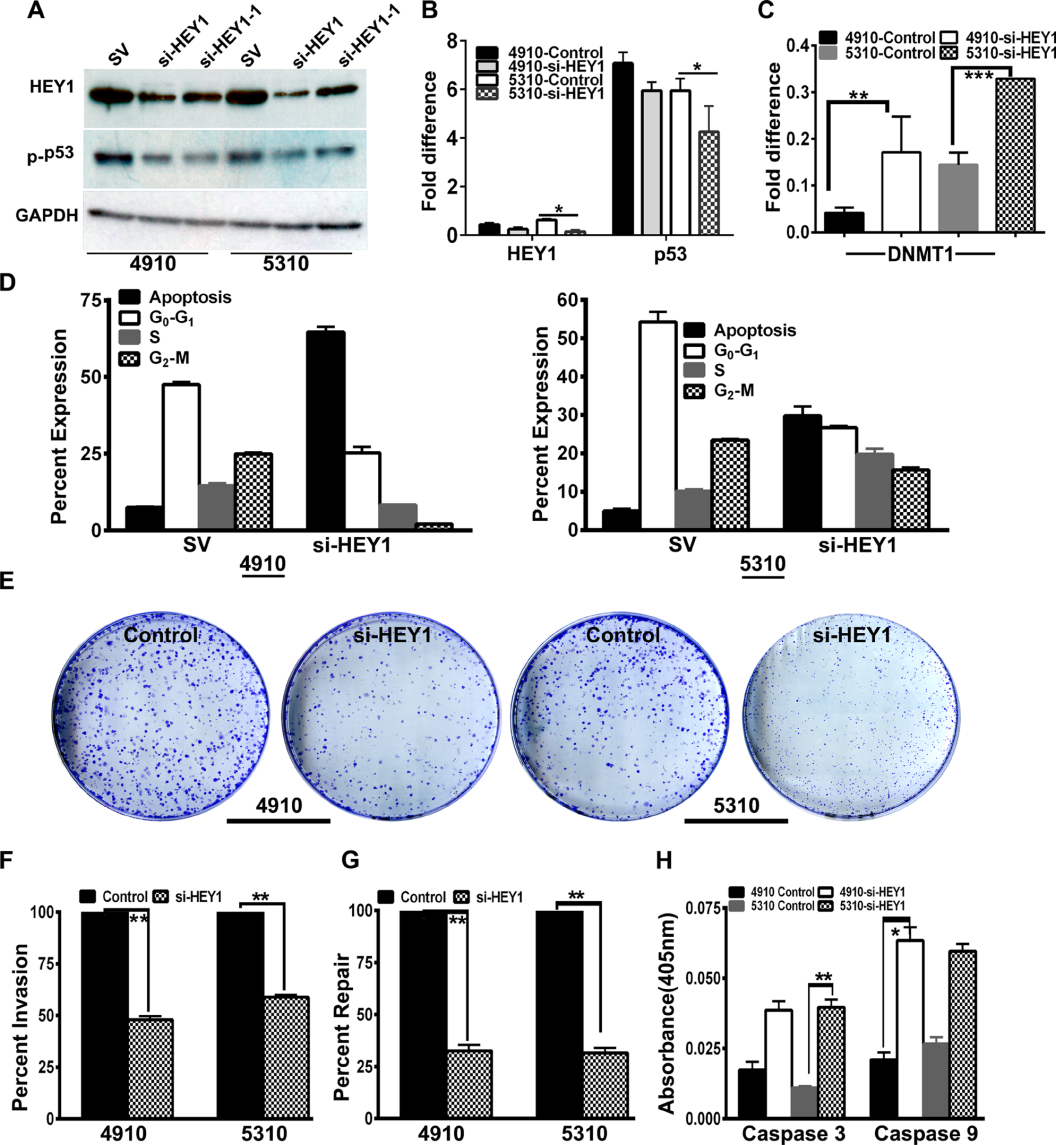
si-HEY1 reduces phenotypes associated with cancer *in vitro* (**A**) Immunoblot analysis of HEY1 and phosphor-p53 levels in SV, si-HEY1 and si-HEY1-1 treatments (SV = Scrambled vector; si-HEY1 = small interfering RNA for HEY1 obtained from pooled sequences; si-HEY1-1 = small interfering RNA for HEY1 obtained from single sequence) (**B** and **C**) qRT-PCR analysis of si-HEY1 treatment to detected the levels of HEY1, p53 and DNMT1 (**D**) FACS analysis (**E**) Clonogenic assay (**F**) Matrigel plug assay (**G**) wound healing assay (**H**) Colorimetric assays to demonstrate caspases 3 and 9 activity (EMD Millipore, Bilerica, MA; kits APT131, APT173).

After observing the aforementioned results following 2.5 mM NaB treatment, we next conducted further experiments by utilizing siRNA approach. Both 4910 and 5310 cells were transfected with siRNA specific to HEY1 (siRNA (si-HEY1) – pooled sequences; siRNA-1 (si-HEY1-1) – single sequence) and scrambled vector (SV). Immunoblot analysis conducted on the lysates of scrambled vector- and siRNA transfected cells showed reduced levels of both HEY1 and phospho-p53 in si-HEY1- treated cells. The pooled siRNA construct of HEY1 was effective when compared to single siRNA sequence construct, in decreasing the HEY1 expression. So, the pooled si-HEY1 construct is thus used in all the experimental conditions (Figure 4A). qPCR analysis confirmed our western blotting results (Figure 4B). Interestingly, DNMT1 gene expression was increased in si-HEY1 treatment (Figure 4C), suggesting that HEY1 can be an epigenetic target in studying glioma progression. The results obtained from FACS analysis on treatment with si-HEY1 were comparable to the results obtained from NaB treatment. We observed that si-HEY1- treated cells showed increased apoptosis (Figure 4D). To address if si-HEY1 treatment was associated with GBM proliferation, we conducted clonogenic assays on both 4910 and 5310 GBM lines. si-HEY1 treatment greatly reduced the colony formation (Figure 4E). Next, using matrigel plug invasion assay, si-HEY1 treated cells demonstrated significant reduction in the invasion abilities of cell lines 4910 and 5310 compared to their respective controls (Figure 4F). Further, using wound healing assay, following the treatment of si-HEY1, we demonstrated that migratory abilities were impaired greatly in both 4910 and 5310 (Figure 4G). Finally, using caspase 3/9 activity assay kit, we observed significant increase in caspase 3 activity levels in the 5310 cell line (*P* ≤ 0.05), and increased caspase 9 activity (*P* ≤ 0.01) in the 4910 cell line (Figure 4H).

### HEY1 interacts with plethora of transcription factors

**Figure 5:**
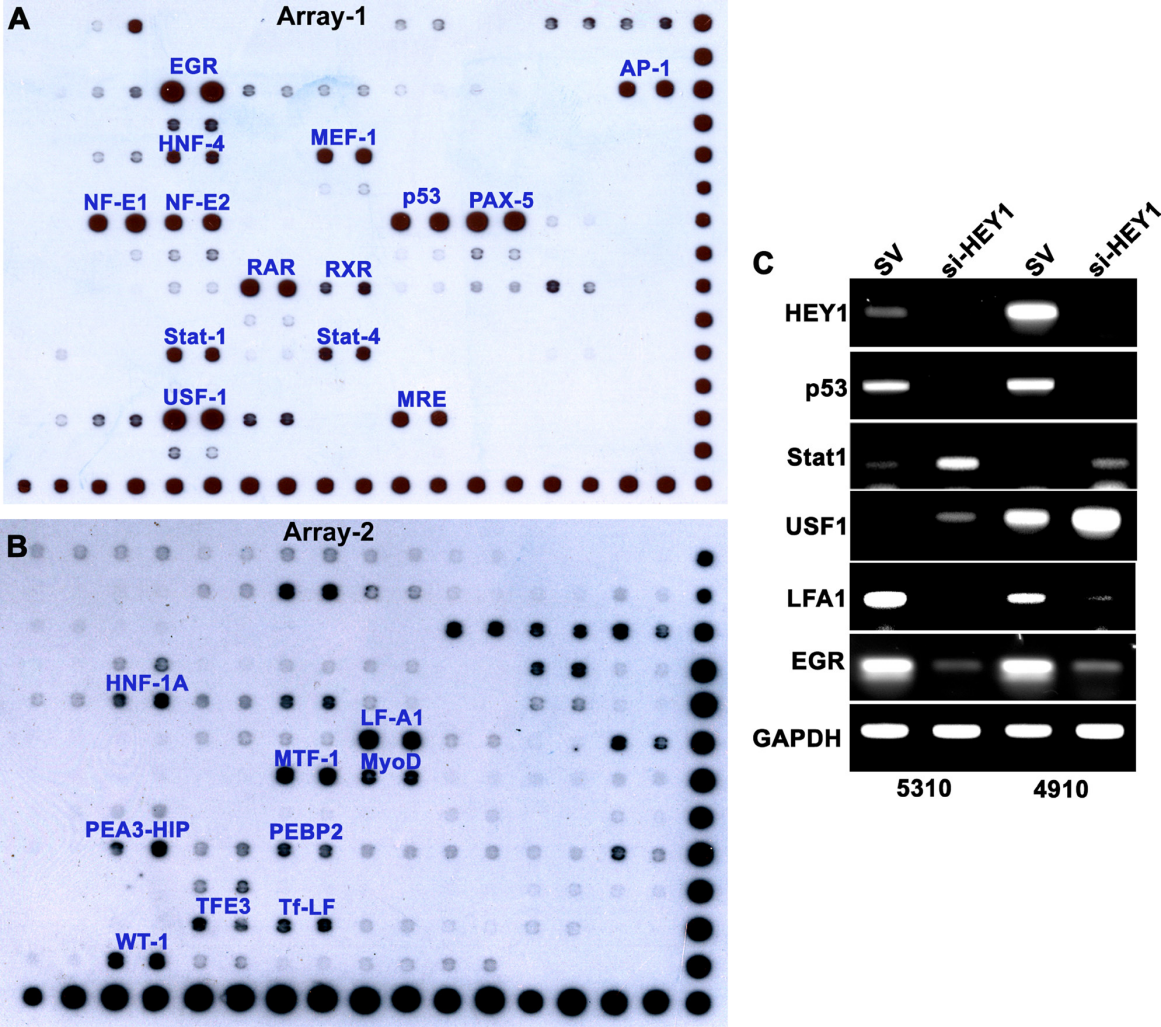
Potential interaction of HEY1 with multiple transcription factors (**A**) TF-TF array was performed using recombinant HEY1 and anti-HEY1 antibody as per the manufacturer's instructions (Affymetrix, Santa Clara, CA; #MA5012) and potential HEY1-interacting proteins are highlighted (**B**) To determine the potential binding of HEY1 with other nuclear proteins and their consensus DNA binding sequences, TF-DNA array was performed according to the manufacturer's instructions (Affymetrix, Santa Clara, CA; #MA1210). The potential HEY1 binding TFs are highlighted (**C**) Confirmatory PCR to evaluate a number of HEY1-associated transcription factors.

To further our understanding of the epigenetic regulation of HEY1 expression and to identify different transcription factors (TFs) that may behave as putative HEY1- interacting partners, we used recombinant HEY1 protein (1μg/μl) and applied them to the TF/DNA arrays. By using two separate TF-TF and TF/DNA arrays we identified unique HEY1 associated DNA binding TFs (54 TFs in array-1 and 96 TFs in array-2). In TF-TF array-1, we found TFs: EGR, AP1, NFE1, p53, PAX5, RAR, USF1 and MRE to be bound to HEY1 (Figure 5A). Additionally, in TF/DNA array-2 we found TFs: LFA1, MTF1, HIP and WT1 (Figure 5B) DNA binding activity to HEY1. We next attempted to validate these findings using PCR analyses. cDNA obtained from scrambled vector and si-HEY1 transfected cells showed that silencing of HEY1 reduced the expression of p53, EGR and LFA, while we observed increased expression levels of STAT1 and USF1 (Figure 5C).

## DISCUSSION

Glioblastoma (GBM) is one of the most difficult-to-treat cancers with no significantly efficacious treatment available. Even as surgical and chemotherapeutic strategies for treating the disease improve, the median survival time from diagnosis for GBM patients has remained at just over one year [22]. Therefore, it is essential to identify new targets, that are both predictive of outcome and can be utilized in novel chemotherapeutics for patients of the disease. In the current study, HEY1, a molecule that mediates Notch signaling, [23] is found to be highly expressed in glioblastoma samples across multiple studies and appears to have a significant role in the proliferative and invasive abilities of GBM. Our immunoblot analysis demonstrated expression of high HEY1 levels across GBM patient samples and an absence of HEY1 in normal brain. This finding was then confirmed in immunohistochemical staining, which showed that within pathological slides of resected GBMs, HEY1 is present in tumor tissue. Since HEY1 was confirmed as highly expressed across GBM patients and not in normal brain; we then set out to evaluate the effectiveness of HEY1 as a potential target for therapy.

**Figure 6:**
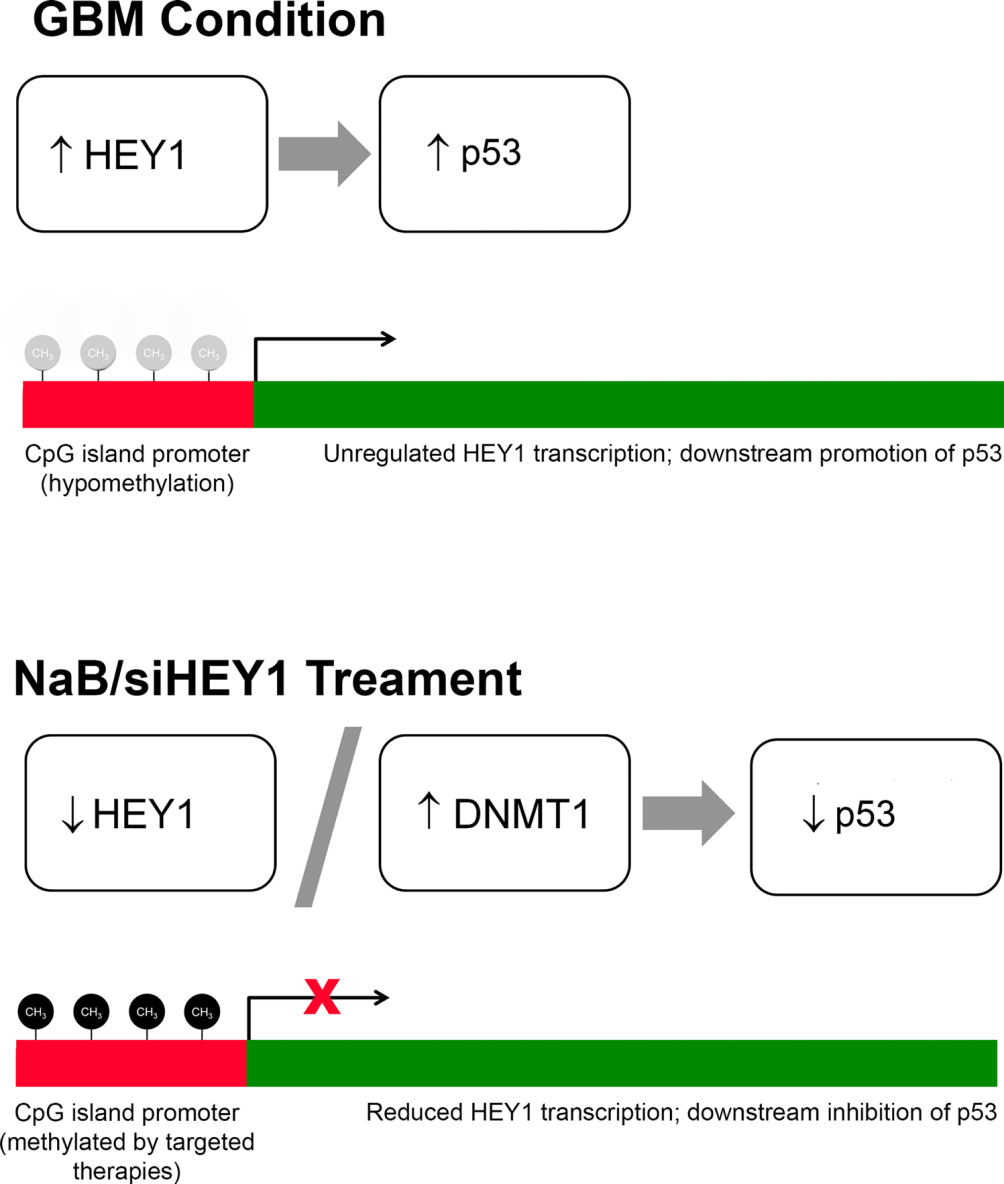
HEY1's methylation status determines the expression levels of p53 and DNMT1 (Top) The illustration entitled “GBM Condition” indicates the untreated status of the HEY1 gene in GBM patients. Hypomethylation of the promoter region (depicted by transparent methyl caps) of the gene in turn causes unregulated transcription of HEY1, precipitating an increase in p53 expression. (Bottom) The illustration entitled “NaB/siHEY1 Treatment” suggests that in both NaB and siRNA treatments of GBM cell lines, methyl caps are now back in place (denoted as black methyl caps) on the promoter region of HEY1. This re-methylation leads to a concurrent increase in DNMT1 expression and decrease in HEY1 expression, ultimately precipitating a decrease in p53 expression.

Additional analysis via bisulfite nucleotide sequencing showed high levels of methylation on CpG islands within the promoter region of HEY1 in a control brain sample, whereas demethylation of these islands within the promoter region appears to occur across samples in GBM tissue. This demethylation, which mechanistically leads to higher transcriptional activity, might explain the presence of HEY1 observed on immunohistochemistry and immunoblot analysis of the biopsy specimens. These findings invite investigation into the efficacy of targeting the methylation status in potential therapies in GBM. Initial findings indicate that the methylating agent sodium butyrate works to re-methylate the CpG-rich promoter sequence of the HEY1 gene (Figure 2C). However, the mechanism of NaB methylation on the regulation of HEY1 expression is more complex than it might initially seem, since it involves other effector molecules and downstream genes in HEY1 signaling. It is known that sodium butyrate is shown to suppress proliferation and promote apoptosis [24]. Specifically, we wanted to study if NaB treatment can reduce HEY1 and its associated genes’ expression within GBM cell lines, and whether or not this reduction could lead to a reversion of cancer phenotypes (Figure 6). Two different cell lines, 4910 and 5310, were used to test the efficacy in the targeting of HEY1 in GBM. Using qRT-PCR, we demonstrated that 2.5 mM NaB and si-HEY1 treatments both increase the levels of DNA Methyl Transferase 1 (DNMT1) in GBM cell lines. DNMT1's predominant role in human genetic activity is to methylate CpG dinucleotides such as those found in the promoter region of HEY1 [25]. Our findings suggest NaB-mediated inactivation of HEY1 in GBM, implicating DNMT1 as a possible effector in the targeting of HEY1.

Although DNMT activity is notably altered by NaB and si-HEY1 in our studies, it may not be the only mechanism by which NaB/si-HEY1 therapies work to inhibit HEY1. Immunoblot analysis indicated that 2.5 mM NaB treatment reduces the expression of HEY1 and p53. Immunofluorescence experiment confirmed the nuclear co-localization of HEY1 and phospho-p53 in 4910 and 5310 cells, whereas NaB- treatment reduced their expression and co-localization in the nucleus. These findings are significant when linked to FACS analysis, that show increased percentages of cells within the apoptotic stages of the cell cycle, suggesting that apoptotic activity might be mediated in part by HEY1/p53 inactivation. si-HEY1 treatment showed similar findings, as indicated by immunoblot analysis, qRT-PCR, and classical assays that assess the hallmarks of cancer. Consistent HEY1 and p53 inhibition and DNMT1 activation by both siHEY1 and NaB treated cells suggest that these effects are more than the consequences of pleotropic action by NaB, but rather due to specific effects on these three genes. Finally, the DNA/protein transcription factor interaction array experiment revealed that HEY1 binds a large number of transcription factors, including both STAT1 and STAT4. Although PCR data does not show si-HEY1 to have a significant effect on STAT1, previous research indicates the overexpression of STAT1 is unique to glioblastoma samples when compared to normal brain tissue samples [26]. More significantly, STAT1 levels have been correlated with shorter median survival time in patients with GBM (STAT1+ = 13 months; STAT1- = 21 months) [31]. Similarly, PAX5 proteins are thought to possibly inhibit tumor cell apoptosis, augmenting tumorigenesis [27]. Furthermore, p53 contains a PAX binding site which serves to regulate the transcriptional inhibition of p53. The significance of these arrays reiterates the number of downstream effects that HEY1 can have as a transcriptional repressor on downstream genes that might all contribute to the development of GBM traits related to its aggressive and treatment-resistant nature.

In summary, we confirm that HEY1 is overexpressed in the GBM condition; DNMT1's reduced activity might lead to hypomethylation of the HEY1 promoter sequence, causing constitutive expression of HEY1 and mutant p53; and therapies that inactivate HEY1 via transcriptional interference or methylation of the CpG island-rich promoter sequence show promise in reducing the cancer phenotype. These three findings indicate that further investigation of HEY1 methylation is warranted as it relates to novel glioblastoma treatments.

## MATERIALS AND METHODS

### Antibodies and reagents

Antibodies for HEY1, p53, p-p53 and GAPDH; small interfering RNA (siRNA - pooled) for HEY1 were purchased from Santa Cruz Biotechnology (Santa Cruz, CA). In this manuscript, we used a pooled siRNA for HEY1 from Santa Cruz Biotechnologies (sc-37914). Additionally, we generated HEY1 siRNA target sequence (single) using pSilencerTM 4.1-CMV (Ambion, Austin, TX), plasmid vector according to the manufacturer's instructions. The target sequences used are si-HEY1-F: GATCCCTGTAGTTAACTCCTCCCTTTCAAGAGAA GGGAGGAGTTAACTACAGTTTTTGGAAA and si-HEY1-R: GGACATCAATTGAGG AGGGAAAGTTCT CTTCCCTCCTCAATTGATGTCAAAAAACCTTTT CGA. Sodium butyrate was obtained from Sigma (St. Louis, MO). All the primers in the study were ordered from IDT. Caspase 3 and 9 kits were bought from Millipore (St. Charles, MO). TF-DNA arrays (Cat # MA5012) and TF-TF arrays (MA1210) were obtained from Affymetrix (Cleveland, OH).

### Growth conditions

Both 4910 and 5310 cells were a kind gift from Dr. David James to the University of Illinois College of Medicine at Peoria. These cells were grown in RPMI-1640 medium supplemented with 10% FBS and 1% penicillin-streptomycin at 37°C.

### RNA expression by Oncomine™ database

The Oncomine^™^ research edition (v4.5) was utilized to investigate the HEY1 mRNA expression profiles in a variety of glioblastoma transcriptomic data sets. In this study, we considered grade IV glioblastoma tumors and the following cut-offs were applied in a pre-filtering step: *p*-value ≤ 0.05 (*t*-test) and fold change (FC) ≥ 2.0.

### Immunoblot analysis

Cell lysates from various treatments were carried out using equal amounts of protein, then resolved via SDS-PAGE. Blots were incubated with 1:500 dilutions of HEY1 and p53 primary antibodies, and subsequently incubated with 1:1000 dilution of species-specific, horseradish peroxidase (HRP)-conjugated secondary antibody. Subsequently, imaging and data analysis was carried out using chemi-luminescence ECL Western blotting detection reagents on Hyperfilm-MP autoradiography film. GAPDH antibody was used to verify equal loading of proteins in all lanes.

### Immunohistochemical analyses of hGBM (human glioblastoma patient) specimens

Normal human brain and hGBM surgical biopsy specimens were obtained from Saint Francis Medical Center (Peoria, IL) and processed in accordance with the UICOMP Institutional Review Board–approved protocols (Protocol #85193). Representative sections of GS-16 and normal human brain specimen were DAB stained with HEY1 antibody as described previously. For DAB immunohistochemistry, sections were probed with the aforementioned antibodies and then stained with DAB and further stained with Hematoxylin. Negative controls were maintained without primary antibody by using IgG fraction. The sections were then visualized using a confocal microscope according to standard protocols.

### Bisulfite modification of genomic DNA and bisulfite sequencing analysis

Genomic DNA was isolated from nine selected glioblastoma tumors and, a normal human brain control. Simultaneously, we isolated genomic DNA from both 5310 and 4910 cell lines using DNeasy tissue kit (Qiagen). Bisulfite reactions were carried out on 5 μg of genomic DNA obtained using EpiTect bisulfite kit (Qiagen) as per the supplied protocol.

### Quantitative real-time PCR (qRT-PCR)/PCR analysis

Total RNA from various experiments was extracted using TRIzol reagent (Life Technologies, Carlsbad, CA), and a cDNA synthesis kit (Roche, Indianapolis, IN) was used to synthesize the necessary cDNA. A CFX96 machine (Bio-Rad, Hercules, CA) was used to complete the qPCR reaction. The gene expression levels were normalized to expression levels of GAPDH. Dissociation curves were evaluated for primer fidelity, and only threshold cycles below 35 cycles were reported. The primers (Integrated DNA Technologies, Chicago, IL) used for this study are listed in Supplementary Table 1.

### Cell cycle analysis via fluorescence-activated cell sorting (FACS)

NaB/siHEY1 treated cells and their respective control 4910/5310 cells were harvested by trypsinization and stained with propidium iodide (PI) (2 mg/mL; Biosure). Suspensions of 1 × 10^6^ cells were analyzed by FACS Caliber System (Becton Dickinson Bioscience) with laser excitation at 488 nm and emission at 639 nm. The percentage of cells in the various phases of the cell cycle (G0/G1, S, and G2/M) was assessed using Cell Quest software (Becton Dickinson Bioscience).

### Cell proliferation assay

Both 4910/5310 cell growth was assayed using the 3-(4, 5-dimethylthiazol-2-yl)-2, 5-diphenyl tetrazolium- bromide (MTT) assay. Cells were incubated with vehicle and sodium butyrate (2.5 mM and 5 mM), for 24 h. Around 100 μl MTT reagent (Invitrogen; Carlsbad, CA) was added to each well and the plates were incubated for 2 h at 37°C to allow MTT to form formazan crystals by reacting with metabolically active cells. Medium was replaced with 100 μl dimethylsulfoxide (DMSO) and plates were incubated for 30 minutes at room temperature with shaking. The optical density was measured at 550 nm.

### Matrigel-plug invasion assay

Matrigel invasion assay was performed by following a previously established protocol [28]. In brief, 1 × 10^6^ control or siHEY1-transfected 5310 and 4910 cells were plated onto Matrigel-coated transwell inserts that were placed in a 12-well plate containing complete medium. After 24 hours of incubation, lower invaded cells were fixed and stained with HEMA-3. The non-invaded cells in the upper chamber were washed using a cotton swab to enable a clear view of the invaded cells. Images of the invaded cells were taken under a light microscope (Olympus IX-71).

### Clonogenic assay

Single cell suspension of around 1000, control and siHEY1 transfected 4910 and 5310 cells were plated in 60 mm plates, spread well, and incubated for 3 weeks until cells in control plates had formed sufficiently large colonies. All the experimental plates were stained with HEMA-3 for 10 min, and colonies were counted and graphically represented.

### Wound repair assay

For the wound healing migration assay, cells were grown to full confluence in a 6-well plate, forming a monolayer. A straight scratch was made manually in individual wells using a 200 μL pipette tip, as described previously [29]. This point was considered the “0 h” and the width of the wound was immediately photographed under the light microscope. The cells were further incubated for about 12–16 h and observed for percentage cell migration.

### Determination of activity of caspase-3 and caspase-9

Caspase-3 and caspase-9 activity of cells were measured using kits purchased from Millipore (Kankakee, IL). 4910, 5310 control and siHEY1 transfected cells were extracted and enzymatic activities of caspase- 3 and -9 were measured using Caspase-3 Colorimetric Assay Kit and Caspase-9 Colorimetric Assay Kit respectively (Millipore) according to manufacturer's instructions. The absorbance was measured at 405 nm for caspase-3 and caspase-9, respectively.

### Transcription factor-transcription factor and transcription factor/DNA interaction arrays

A ThermoFisher Affymetrix TF-TF Interaction Array III (Santa Clara, CA) was obtained for this protocol, and methods described in Mukhopadhyay et al. (2006) were utilized in order to carry out the TF-TF binding array and TF-DNA binding array protocols [30]. HEY1-associated transcription factors and DNA fragments were immunoprecipitated in this study.

## SUPPLEMENTARY MATERIALS FIGURES AND TABLES


