# Supplementary material for: Methylation regulates HEY1 expression in glioblastoma
Source: Oncotarget. 2017 May 16;8(27):44398–409. doi: 10.18632/oncotarget.17897 (PMC5546488; doi:10.18632/oncotarget.17897)
Supplement: Supplementary file 1 [file oncotarget-08-44398-s001.pdf]

Methylation regulates HEY1 expression in glioblastoma

Supplementary Materials

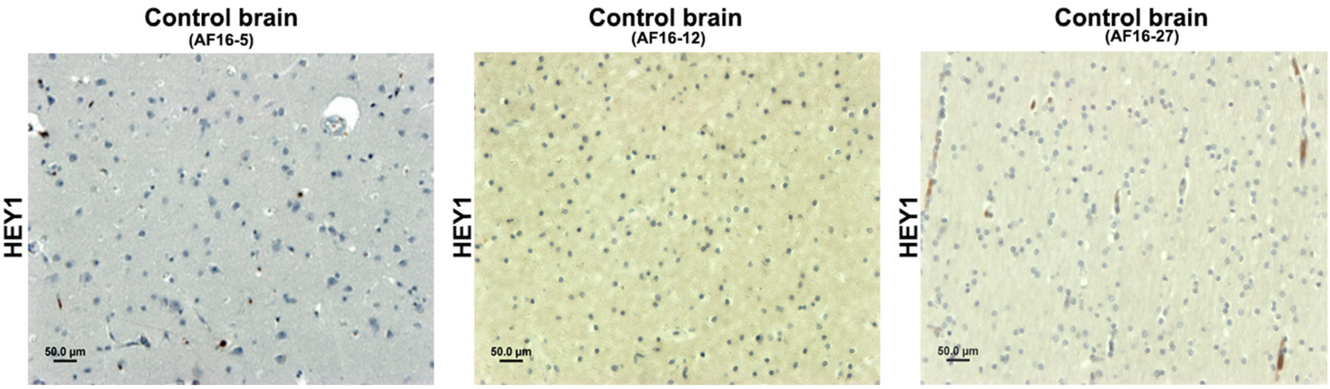

Supplementary Figure 1: HEY1 expression in normal brain samples. Immunohistochemical analysis conducted on three normal brain specimens obtained from autopsies revealed negligible expression of HEY1 ($n = 2$).

Supplementary Table 1: : List of Pimers used in this study

1	HEY1	F	5′-TGGATCACCTGAAAATGCTG-3′
		R	5′-CGAAATCCCAAACTCCGATA-3′
2	P53	F	5′-CCAGGGCAGCTACGGTTTC-3′
		R	5′-CTCCGTCATGTGCTGTCACTG-3′
3	GAPDH	F	5′-AATCCCATCACCATCTTCCA-3′
		R	5′-TGGACTCCACGACGTACTCA-3′
4	DNMT1	F	5′-CCTAGTTCCGTGGCTACGAGGAGAA-3′
		R	5′-TCTCTCTCCTCTGCAGCCGACTCA-3′
5	STAT1	F	5′-CAGCTTGACTCAAAATTCCTGGA-3′
		R	5′-TGAAGATTACGCTTGCTTTTCCT-3′
6	LFA1	F	5′-GATCAACGATTACGTGGAGAAGG-3′
		R	5′-CCTAAACGCTTCATCATAGGCA-3′
7	EGR	F	5′-CCACGCCGAACACTGACATT-3′
		R	5′-GAGGGGTTAGCGAAGGCTG-3′
8	USF1	F	5′-CTGCTGTTGTTACTACCCAGG-3′
		R	5′-TCTGACTTCGGGGAATAAGGG-3′
